# Supplementary material for: Polycyclic aromatic hydrocarbons and their metabolites in bronchoalveolar lavage and urine samples from patients with inhalation injury throughout their hospitalization: A prospective pilot study
Source: PLoS One. 2024 Aug 1;19(8):e0308163. doi: 10.1371/journal.pone.0308163 (PMC11293749; doi:10.1371/journal.pone.0308163)

Supplement

Polycyclic aromatic hydrocarbons in bronchoalveolar lavage and urine samples from patients with inhalation injury throughout their hospitalization: a prospective pilot study

Katerina Vyklicka[1]*, Petr Gregor[1]*, Bretislav Lipovy[2,3], Filip Raska[2], Petr Kukucka[1], Petr Senk[1], Petra Pribylova[1], Pavel Čupr[1], Petra Borilova Linhartova[1#]

Contents

Supplement 1

Polycyclic aromatic hydrocarbons in bronchoalveolar lavage and urine samples from patients with inhalation injury throughout their hospitalization: a prospective pilot study 1

Case series of ten patients with inhalation injury 2

Patient 1 4

Patient 2 5

Patient 3 6

Patient 4 7

Patient 5 8

Patient 6 9

Patient 7 10

Patient 8 11

Patient 9 12

Patient 10 13

Case series of ten patients with inhalation injury

The next pages contain a basic description of each of the ten patients included in our study:

- the characterization of the patients and their medical history throughout hospitalization with the inhalation injury, such as infection occurrence,
- changes in clinical markers during hospitalization,
- changes in polycyclic aromatic hydrocarbons (PAHs) and their metabolites (OH-PAHs) during hospitalization.

Further, the description of each patient contains 3 graphs:

Figure 1. Changes in the clinical markers over time – showing the changes in individual plasma-/serum-based clinical markers throughout hospitalization. The grey boxes indicate that the particular parameter was not measured on the respective day of hospitalization. The white triangles highlight that the value of the marker was above (▲) or below (▼) the physiological range.

Figure 2. Changes in the sum of OH-PAHs in urine over time – showing the kinetic changes of OH-PAHs in urine samples of all patients during the hospitalization, with the particular patient highlighted in red and all others in grey.

Figure 3. Changes in the individual OH-PAHs in urine over time – showing the changes in concentrations of individual OH-PAHs in urine samples throughout hospitalization. The white boxes indicate that the OH-PAHs were not detected in the sample on the particular day of hospitalization, values in the boxes describe the detected concentrations of OH-PAH (ng/ml).

Abbreviations

1-hydroxynaphthalene	1-OH-Naph
2-hydroxynaphthalene	2-OH-Naph
2-hydroxyfluorene	2-OH-Fluo
3-hydroxyfluorene	3-OH-Fluo
1-hydroxyphenanthrene	1-OH-Phen
4-hydroxyphenanthrene	4-OH-Phen
9-hydroxyphenanthrene	9-OH-Phen
2/3-hydroxyphenanthrene	2/3-OH-Phen
1-hydroxypyrene	1-OH-Pyr

Above physiological range	▲
Below physiological range	▼
Below detection limit	□ (white box)
The evaluation was not performed	■ (grey box)

ABSI	abbreviated burn severity index
BAL	bronchoalveolar lavage
CRP	C-reactive protein
PCT	procalcitonin
TBSA	total body surface area

Patient 1

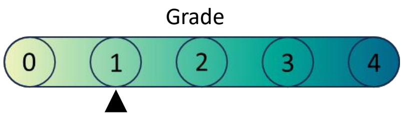

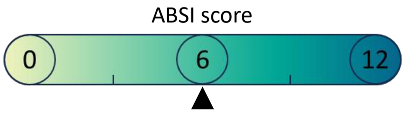

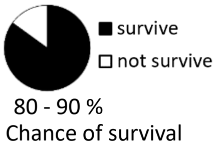

Patient 1 was hospitalized with a mild inhalation injury. ABSI score during the hospitalization was 6 and the chance of survival was between 80 and 90 %. No microbial infections were detected during hospitalization.

All clinical variables showed mostly normal values (see Figure 1 on this page). Notably, CRP, total protein, and albumin were outside the physiological range in the second half of hospitalization.

In BAL, the PAHs such as 6H-benzo[c]chromen-6-one and 9,10-anthraquinone were found in both lungs throughout the hospitalization. Further, 1-nitropyrene and 1,3-dinitropyrene were found in the right lung (data not shown).

In the urine, the high OH-PAHs concentrations from Day 1 decreased over the first three days of hospitalization (see Figure 2 on this page). 1-OH-Naph (15.2 ng/ml) and 2-OH-Naph (29.1 ng/ml) were the most abundant, remaining OH-PAHs were in trace amounts (see Figure 3 on this page).

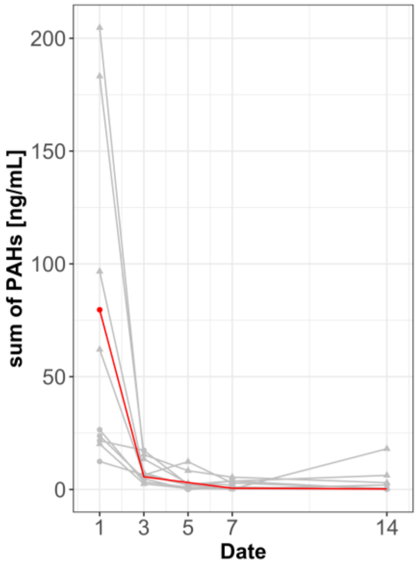

Figure 2. Sum of OH-PAHs in urine

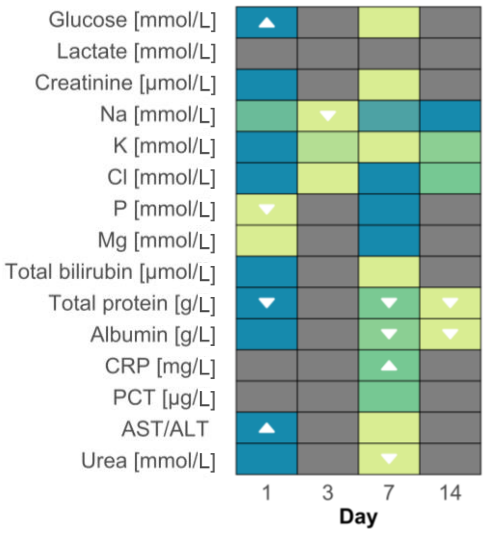

Figure 1. Clinical markers

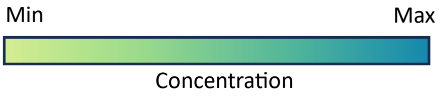

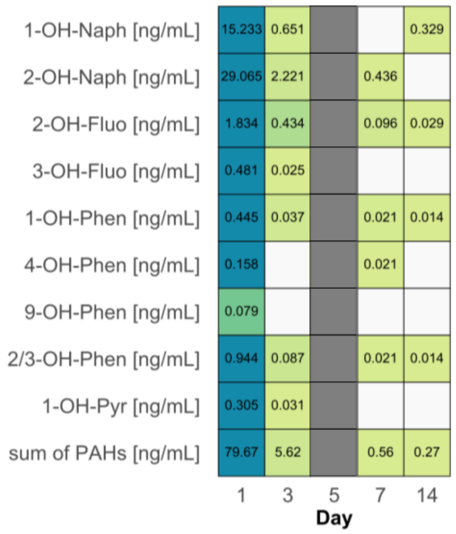

Figure 3. Individual OH-PAHs in urine

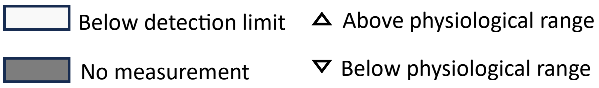

Patient 2

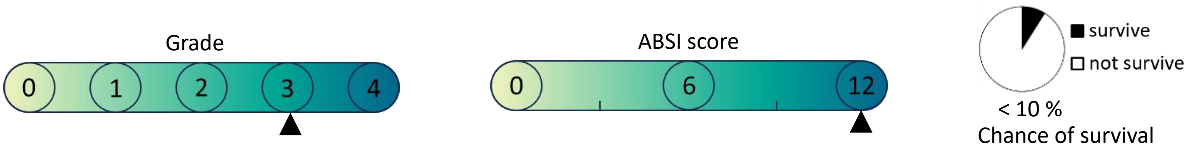

Patient 2 was hospitalized with severe inhalation injury. ABSI score during hospitalization was 12, indicating a chance of survival of less than 10 %. This patient had a broad range of infections, mainly caused by *Klebsiella pneumonia* and *Staphylococcus aureus* but fungal infections (*Metschnikowia* spp., *Saccharomyces* spp., *Alternaria* spp.) were also present. For all those reasons, the patient died after 14 days of hospitalization.

Almost all clinical variables were outside the normal range throughout the entire hospitalization (see Figure 1 on this page).

In the BAL samples, PAHs such as 1-nitropyrene (in low concentration), 6H-benzo[c]chromen-6-one, and 9,10-anthraquinone were detected during the hospitalization (data not shown).

The OH-PAHs were detected in high concentrations in the urine sample from Day 1 of the hospitalization; however, the concentration significantly decreased in the first 3 days of the hospitalization (see Figure 2 on this page). OH-naphthalenes were the most abundant (see Figure 3 on this page).

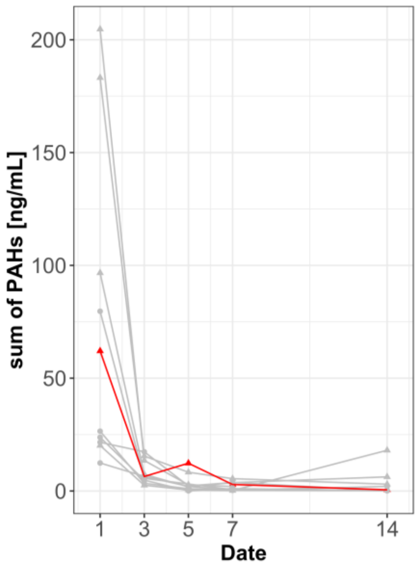

Figure 2. Sum of OH-PAHs in urine

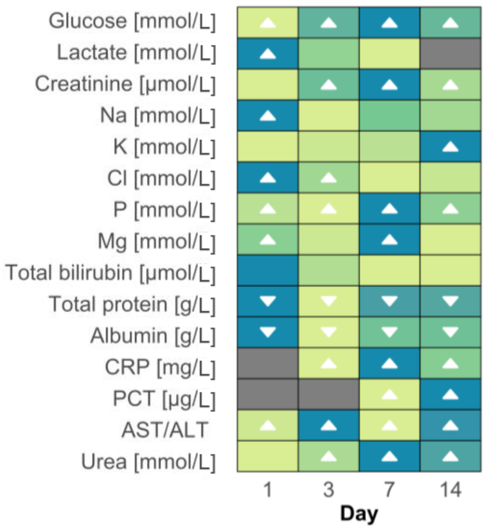

Figure 1. Clinical markers

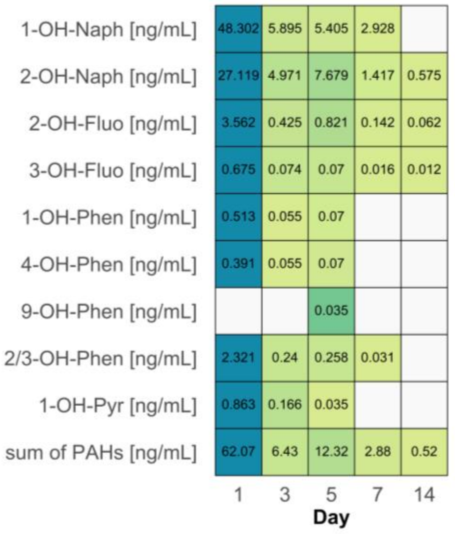

Figure 3. Individual OH-PAHs in urine

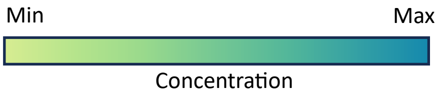

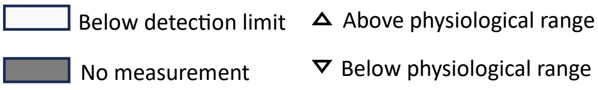

Patient 3

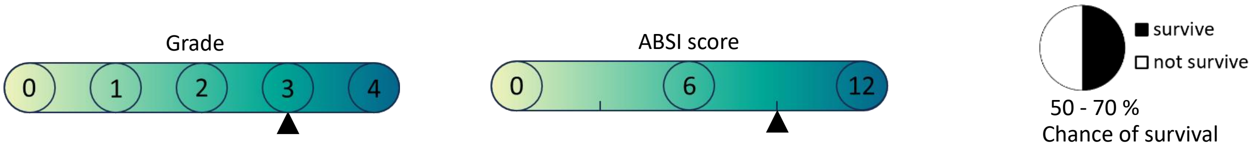

Patient 3 was hospitalized with severe inhalation injury. The ABSI score during hospitalization was 9, with a chance of survival estimated at around 50 %. No microbial infections were detected.

Most clinical variables were within normal limits (see Figure 1 on this page), although CRP and the AST/ALT ratio were high above normal. On the reverse, the total protein and albumin were below normal limits.

Of PAHs, 1-nitropyrene, 3-nitrophenantrene, and 9,10-anthraquinone were detected during the hospitalization in the BAL (data not shown).

In urine, high concentrations of OH-PAHs were found in the samples from Day 1 of the hospitalization; however, the concentration significantly decreased over the first 3 days of the hospitalization (see Figure 2 on this page).

The OH-PAHs sum is one of the highest among all analyzed patient. The highest values were found for 1-OH-Naph (49.7 ng/ml) and 2-OH-Naph (40.6 ng/ml) (see Figure 3 on this page).

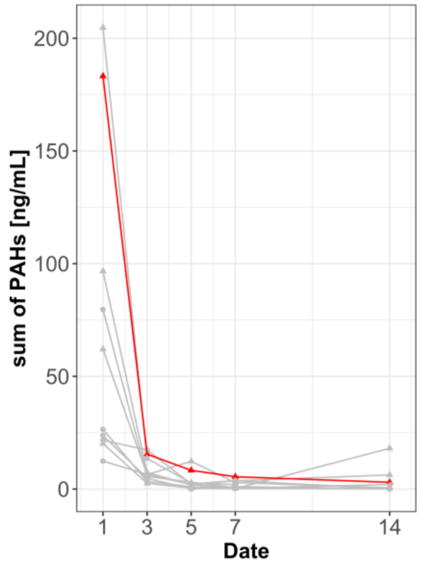

Figure 2. Sum of OH-PAHs in urine

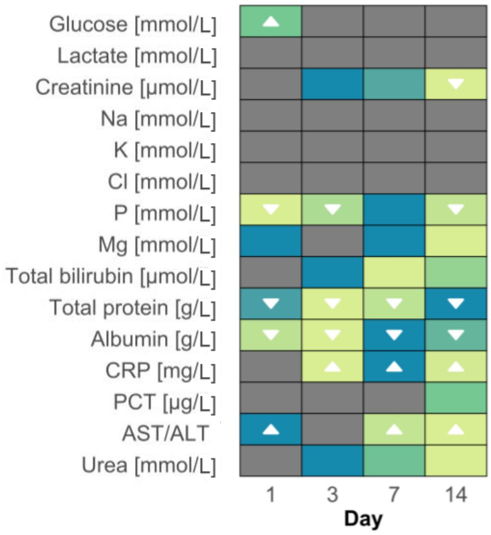

Figure 1. Clinical markers

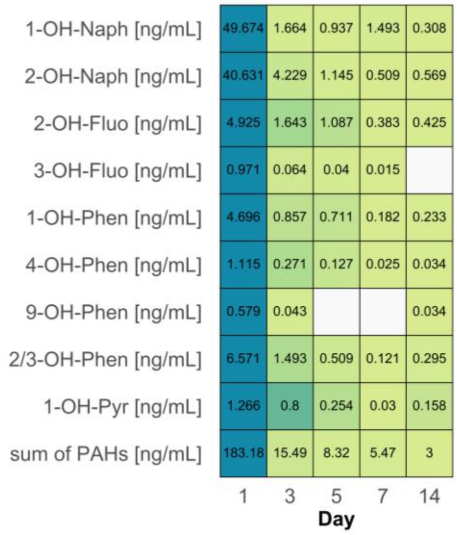

Figure 3. Individual OH-PAHs in urine

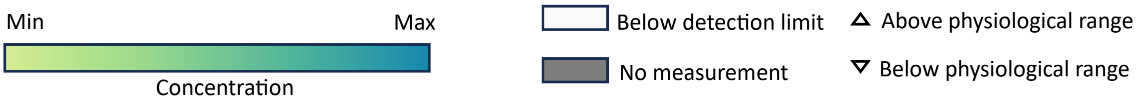

Patient 4

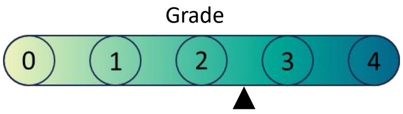

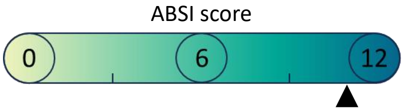

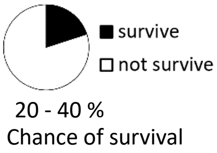

Patient 4 was hospitalized with inhalation injury classified between moderate and severe. ABSI score during hospitalization was 11, with the chance of survival between 20 and 40 %. No microbial infections were detected. The patient died on Day 7 of hospitalization.

All clinical variables left the physiological range during hospitalization (see Figure 1 on this page). The patients had a low total protein level. High above-normal values were detected for the AST/ALT ratio throughout the hospitalization.

In BAL samples, PAHs such as 9-nitrophenantrene/3-nitrophenantrene and 1-nitropyrene were found (data not shown).

The OH-PAHs sum in the urine is one of the highest among all analyzed patients (see Figure 2 on this page); however, it decreased to the minimum concentrations in 3 days. The highest values were detected for 1-OH-Naph (92.9 ng/ml), 2-OH-Naph (37.9 ng/ml), 2/3-OH-Phen (19.7 ng/ml), 1-OH-Phen (18.3 ng/ml), and 1-OH-Pyr (11.7 ng/ml) (see Figure 3 on this page).

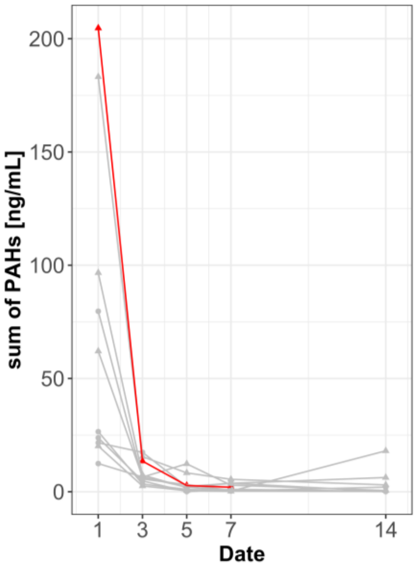

Figure 2. Sum of OH-PAHs in urine

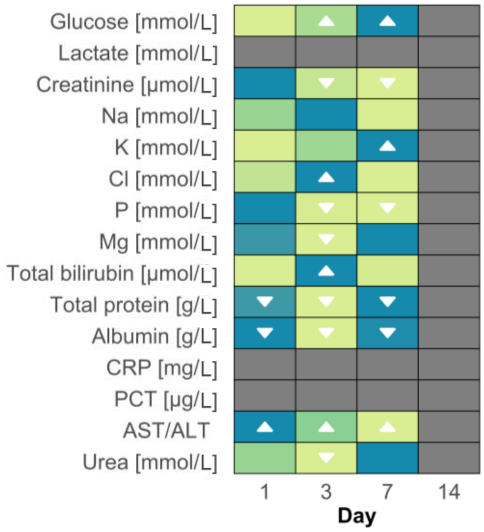

Figure 1. Clinical markers

Day	1	3	5	7	14
1-OH-Naph [ng/mL]	92.933	7.034	1.087	0.671	
2-OH-Naph [ng/mL]	37.872	7.347	2.103	1.341	
2-OH-Fluo [ng/mL]	6.942	1.583	0.386	0.312	
3-OH-Fluo [ng/mL]	2.087	0.293	0.07	0.031	
1-OH-Phen [ng/mL]	18.322	2.755	0.403	0.187	
4-OH-Phen [ng/mL]	3.532	0.606	0.035	0.047	
9-OH-Phen [ng/mL]	0.179	0.039			
2/3-OH-Phen [ng/mL]	19.739	2.638	0.245	0.218	
1-OH-Pyr [ng/mL]	11.711	4.103	0.491	0.265	
sum of PAHs [ng/mL]	204.69	13.51	2.75	1.97	

Figure 3. Individual OH-PAHs in urine

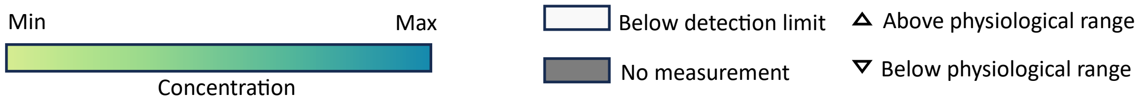

Patient 5

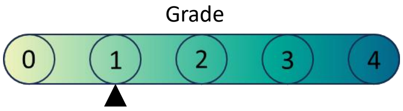

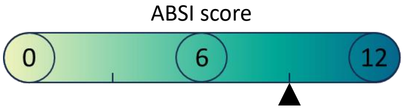

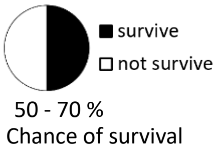

Patient 5 was hospitalized with a mild inhalation injury. The ABSI score during hospitalization was 9, and the chance of survival was between 50 and 70 %. No microbial infections were detected.

The total protein value in this patient was low throughout hospitalization. Further, high values above the physiological limits of AST/ALT and glucose persisted throughout the entire hospitalization (see Figure 1 on this page).

In BAL, PAHs such as 9-nitrophenanthrene, 3-nitrophenanthrene, and 1-nitropyrene were found (data not shown).

The sum of OH-PAHs in the urine was one of the lower among all analyzed patients, it reached the maximum (22.5 ng/ml) first day of hospitalization (see Figure 2 on this page).

The highest concentration was detected for 1-OH-Naph (30.4 ng/ml) and decreased to minimum within three days of hospitalization. The rest of the OH-PAHs is in low or trace amounts (see Figure 3 on this page).

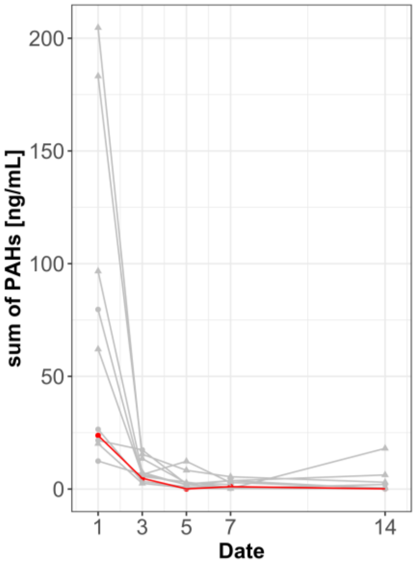

Figure 2. Sum of OH-PAHs in urine

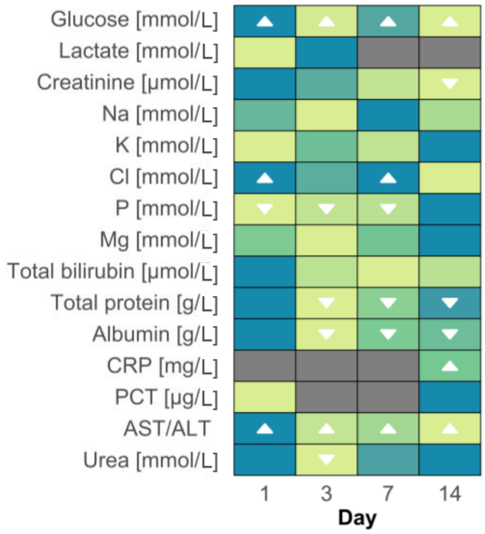

Figure 1. Clinical markers

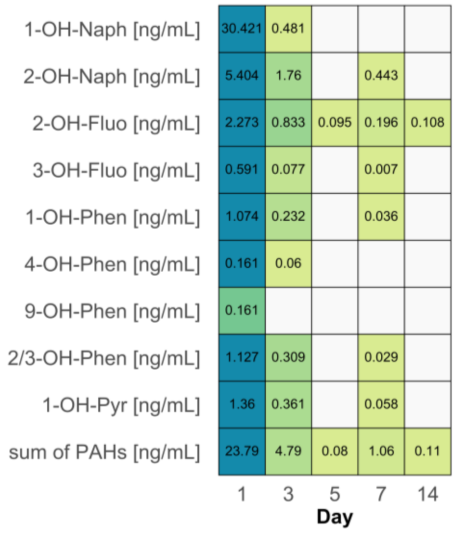

Figure 3. Individual OH-PAHs in urine

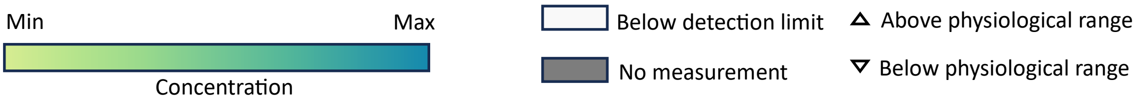

Patient 6

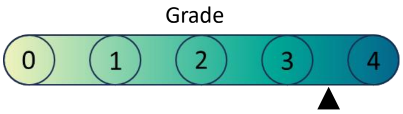

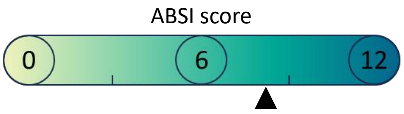

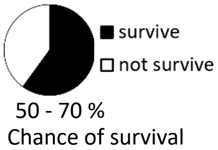

Patient 6 was hospitalized with inhalation injury, which was classified between severe to massive. ABSI score during hospitalization was 8, and the chance of survival was between 50 and 70 %. The patient suffered from a fungal infection caused by *Candida albicans* at the beginning of the hospitalization.

The clinical variables were mostly outside the physiological range; however, they slightly improved at the end of Day 14 of hospitalization (see Figure 1 on this page). Notably, high values above the physiological limits were recorded for bilirubin in the blood and the AST/ALT ratio throughout the hospitalization.

In the BAL samples, mainly 9,10-anthraquinone accompanied by trace amounts of 3-nitrophenanthrene, 6H-benzo[c]chromen-6-one, and 1-nitropyrene were detected even in the later days of hospitalization (data not shown).

In urine, initially high concentrations of the OH-PAHs decreases to a minimum within 3 days of hospitalization (see Figure 2 on this page). 2-OH-Naph (27.8 ng/ml), 1-OH-Naph (22.4 ng/ml), 1-OH-Phen (22.2 ng/ml), and 2/3-OH-Phen (13.2. ng/ml) were the most abundant (see Figure 3 on this page).

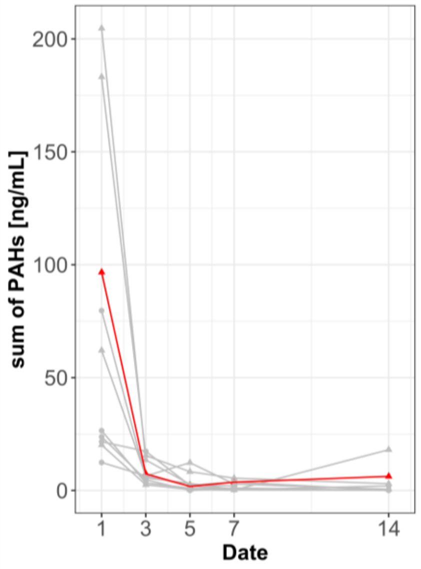

Figure 2. Sum of OH-PAHs in urine

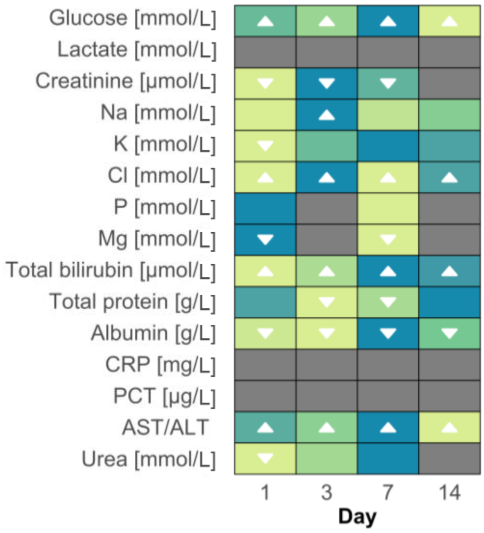

Figure 1. Clinical markers

	Day 1	Day 3	Day 5	Day 7	Day 14
1-OH-Naph [ng/mL]	22.423	2.069	0.442	0.619	0.476
2-OH-Naph [ng/mL]	27.847	3.15	1.192	1.171	1.555
2-OH-Fluo [ng/mL]	3.442	0.553	0.265	0.338	0.603
3-OH-Fluo [ng/mL]	1.043	0.053	0.022	0.011	
1-OH-Phen [ng/mL]	22.215	1.95	1.038	0.743	1.402
4-OH-Phen [ng/mL]	1.262	0.303	0.199	0.18	0.246
9-OH-Phen [ng/mL]	0.302	0.066		0.023	0.025
2/3-OH-Phen [ng/mL]	13.245	0.922	0.486	0.642	0.535
1-OH-Pyr [ng/mL]	9.042	0.659	0.375	0.439	0.484
sum of PAHs [ng/mL]	96.67	7.38	1.82	3.7	6.27

Figure 3. Individual OH-PAHs in urine

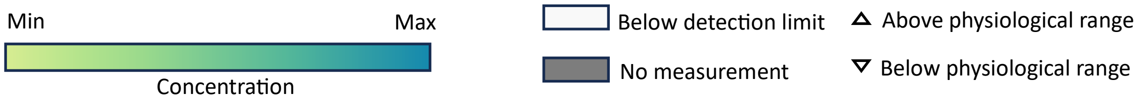

Patient 7

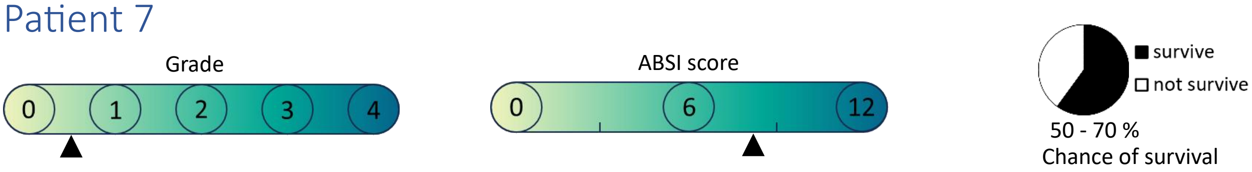

Patient 7 was hospitalized with a very mild inhalation injury; the ABSI score during hospitalization was 8, and the chance of survival was between 50 and 70 %. No microbial infections were detected.

Almost all values of clinical variables were within the physiological limits (see Figure 1 on this page). However, CRP was above the physiological limits on Day 7 of hospitalization and the AST/ALT ratio was above the physiological range throughout the entire hospitalization.

BAL samples contained low amounts of PAHs, with only 3-nitrophenantrene, 9,10-anthraquinone, and 5,12-naphthacenequinone found in the lungs in trace amounts (data not shown).

The BAL findings were in line with the data from urine samples; the concentration of the OH-PAHs was low even on Day 1 of hospitalization. The sum of OH-PAHs was very low (see Figure 2 on this page), with 2-OH-Naph (15.3 ng/ml) being the most abundant metabolite (see Figure 3 on this page).

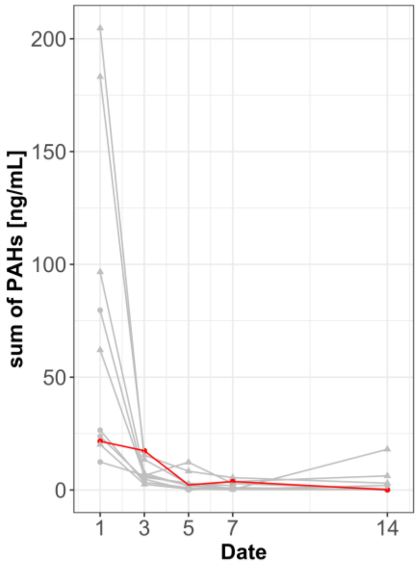

Figure 2. Sum of OH-PAHs in urine

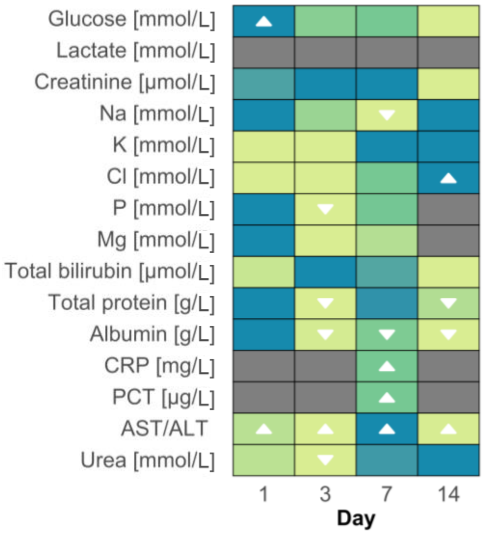

Figure 1. Clinical markers

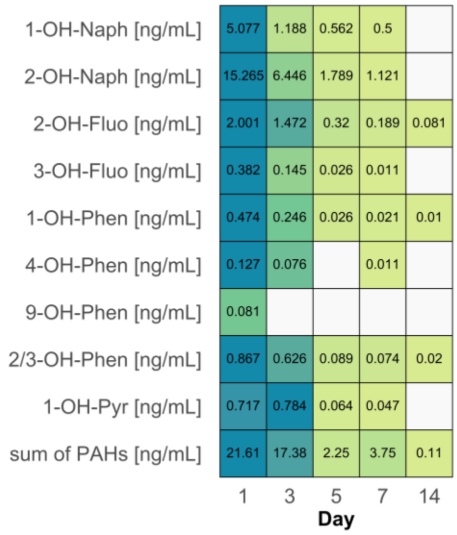

Figure 3. Individual OH-PAHs in urine

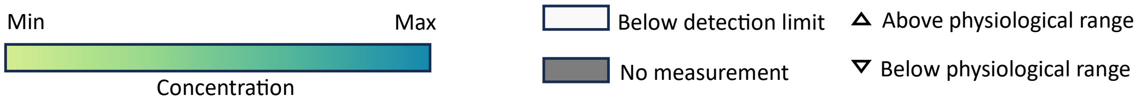

Patient 8

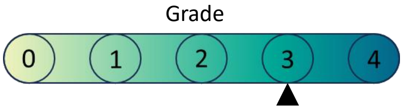

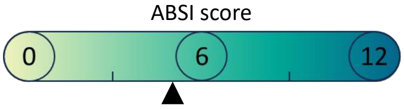

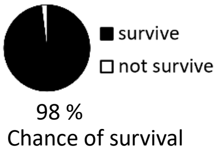

Patient 8 was hospitalized with severe inhalation injury. The ABSI score during hospitalization was 5, and the chance of survival was 98 %. No microbial infections were detected. The patient was deintubated after 3 days of hospitalization.

The patient had high CRP values, which exceeded the physiological range during hospitalization; however, the rest of the clinical variables were mostly within the physiological limits (see Figure 1 on this page).

10 PAHs were detected in BAL, which is the widest range among all patients in the study. However, most of the analytes were in low concentrations (benzo(a)anthracen, chrysene, benzo(ghi)perylene, retene, benzo(ghi)fluoranthene, triphenyl, 3-nitroacenaphthene, 2-nitrofluorene, and 7-nitrobenzoanthracene). The 1,4-naphthoquinone was the most abundant, with almost 110 ng/ml (data not shown).

In the urine, the OH-PAHs were detected in low concentration, sum of OH-PAHs reached only 20.2 ng/ml in maximum (see Figure 3 on this page). However, interestingly the values increase in the urine after day 7 of the hospitalization (see Figure 2 on this page).

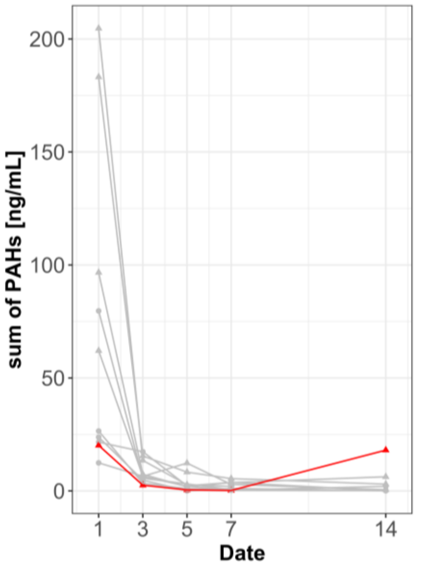

Figure 2. Sum of OH-PAHs in urine

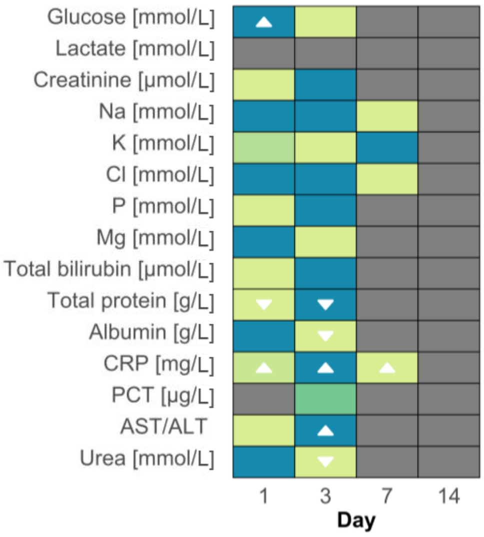

Figure 1. Clinical markers

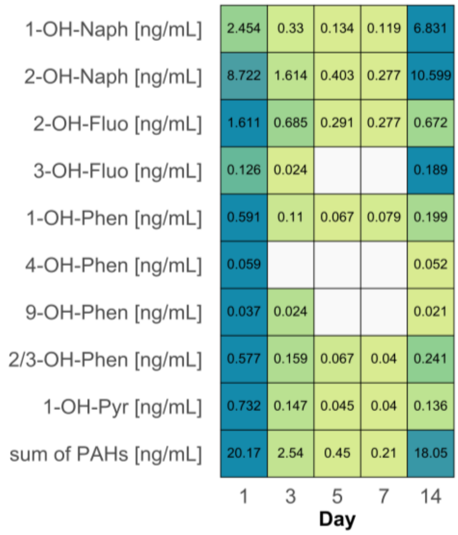

Figure 3. Individual OH-PAHs in urine

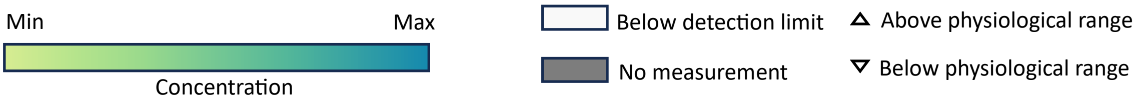

Patient 9

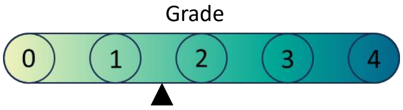

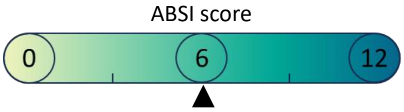

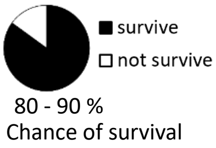

Patient 9 was hospitalized with an inhalation injury classified between mild and moderate. ABSI score during hospitalization was 6, and the chance to survival was between 80 and 90 %. The patient suffered from infections caused by *Haemophilus influenzae* and *Enterobacter cloacae* during hospitalization.

The patients had high CRP values throughout the hospitalization. On the other hand, total protein values at the beginning of the hospitalization were below the physiological range (see Figure 1 on this page).

Of PAHs, mainly 1,4-naphthoquinone was detected in the BAL samples, with a high concentration reaching 150 ng/ml. Further, benzo(a)anthracene, chrysene, and triphenyl were found in this patient during thehospitalization (data not shown).

The concentration of OH-PAHs in the urine throughout the whole hospitalization was low (see Figure 2 on this page). The sum of OH-PAHs on Day 1 was only 12.4 ng/ml (see Figure 3 on this page).

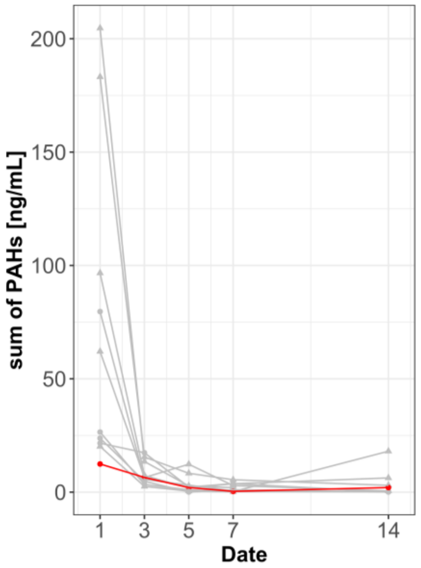

Figure 2. Sum of OH-PAHs in urine

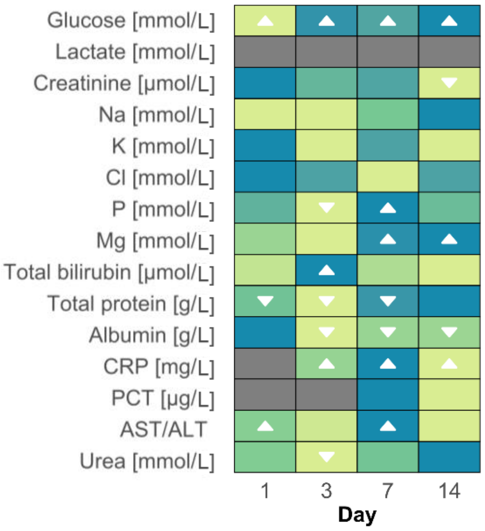

Figure 1. Clinical markers

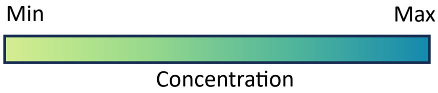

Day	1	3	5	7	14
1-OH-Naph [ng/mL]	1.281	0.442	0.317	0.068	0.876
2-OH-Naph [ng/mL]	6.268	2.639	1.734	0.313	0.695
2-OH-Fluo [ng/mL]	0.944	0.552	0.113	0.041	0.094
3-OH-Fluo [ng/mL]	0.075	0.025	0.011		0.017
1-OH-Phen [ng/mL]	0.195	0.055	0.023	0.027	0.017
4-OH-Phen [ng/mL]	0.022	0.012			
9-OH-Phen [ng/mL]	0.022	0.012	0.011		
2/3-OH-Phen [ng/mL]	0.232	0.117	0.034	0.027	0.026
1-OH-Pyr [ng/mL]	0.262	0.123	0.057		0.009
sum of PAHs [ng/mL]	12.43	6.48	2.03	0.35	2.02

Figure 3. Individual OH-PAHs in urine

Below detection limit
Above physiological range
No measurement
Below physiological range

Patient 10

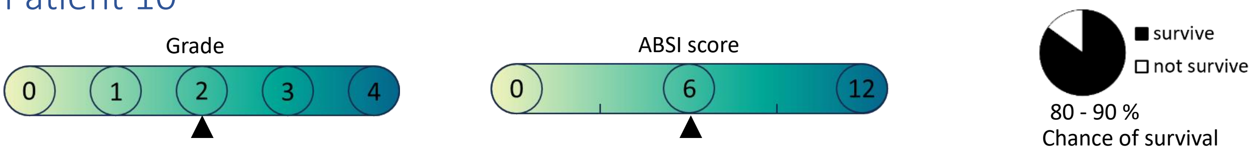

Patient 10 was hospitalized with moderate inhalation injury. ABSI score during hospitalization was 6, and the chance of survival ranged between 80 and 90 %. The patient had an infection caused by *Enterobacter cloacae* and *Pseudomonas aeruginosa* at the end of his hospitalization.

The patient had high values, above the physiological range, of urea and the AST/ALT ratio in the blood. On the other hand, low levels of albumin were found in the blood (see Figure 1 on this page).

PAHs such as benzo(a) anthracene, chrysene, benzo(ghi)perylene, triphenyl, 7-nitrobenzoanthracene, and 6-nitrochrysene were found in the BAL samples of this patient in low concentrations (data not shown).

The concentration of OH-PAHs in urine was low during the whole hospitalization (see Figure 2 on this page); the highest sum of OH-PAHs was on Day 1 of hospitalization (26.5 ng/ml), with 1-OH-Naph (9.6 ng/ml) and 2-OH-Naph (10 ng/ml) being the most abundant of all analyzed OH-PAHs in this patient (see Figure 3 on this page).

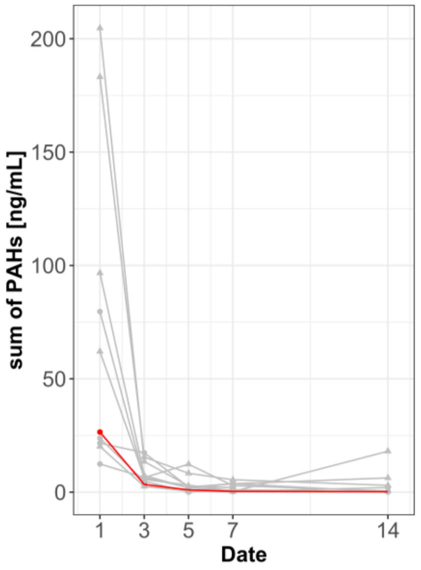

Figure 2. Sum of OH-PAHs in urine

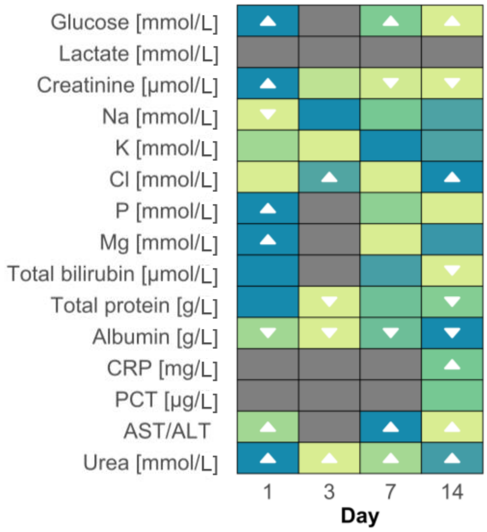

Figure 1. Clinical markers

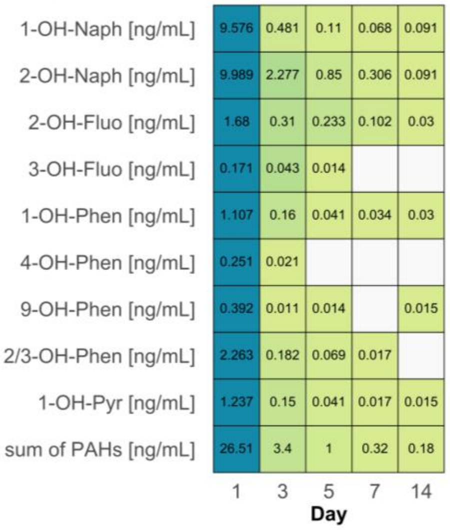

Figure 3. Individual OH-PAHs in urine

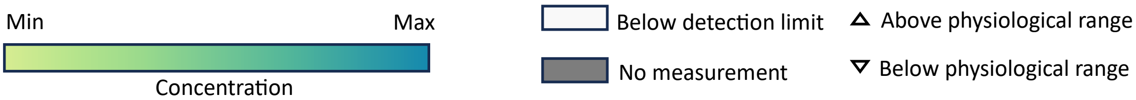

Supplement: S1 File — Nitro-PAHs information (MRM, RT, linearity, calibration range). Oxy-PAHs information (MRM, RT, linearity, calibration range). Method performance parameters (% recovery average, % RSD). List of used chemicals and their purity. PAHs, oxy-PAHs, nitro-PAHs LOD and LOQ values in BAL samples. OH-PAHs LOD and LOQ values in urine samples. (PDF) [file pone.0308163.s002.pdf]
