# Supplementary material for: Polycyclic aromatic hydrocarbons and their metabolites in bronchoalveolar lavage and urine samples from patients with inhalation injury throughout their hospitalization: A prospective pilot study
Source: PLoS One. 2024 Aug 1;19(8):e0308163. doi: 10.1371/journal.pone.0308163 (PMC11293749; doi:10.1371/journal.pone.0308163)
Supplement: S2 File — The supplementary data contain a basic description of each of the ten patients included in our study: i) the characterization of the patients and their medical history throughout hospitalization with the inhalation injury, such as infection occurrence, ii) changes in clinical markers during hospitalization, and iii) changes in polycyclic aromatic hydrocarbons (PAHs) and their metabolites (OH-PAHs) during hospitalization. (PDF) [file pone.0308163.s003.pdf]

Supplementary Tables

Contents

Supplementary Tables 1

Abbreviations 1

Table S1. PAHs information (SIM, RT, linearity, calibration range) 2

Table S2. Nitro-PAHs information (MRM, RT, linearity, calibration range) 3

Table S3. Oxy-PAHs information (MRM, RT, linearity, calibration range) 4

Table S4. Method performance parameters (% recovery average, % RSD) 5

Table S5. List of used chemicals and their purity 6

Table S6. PAHs, oxy-PAHs, nitro-PAHs LOD and LOQ values in BAL samples 7

Table S7. OH-PAHs LOD and LOQ values in urine samples 8

Abbreviations

BAL	BronchoAlveolar Lavage
LOD	Limit of Detection
LOQ	Limit of Quantification
MRM	Multiple Reaction Monitoring
RT	Retention Time
SIM	Selected Ion Monitoring

Table S1. PAHs information (SIM, RT, linearity, calibration range)

Analyte	Quant SIM	Qual SIM1	Qual SIM2	RT	linearity (R^2)	calibration range
	m/z	m/z	m/z	min		ng/mL
D8-naphtalene	136	137	134	9.03	0.999	1-1000
naphtalene	128	129	126	9.06	0.999	1-1000
biphenyl	154	153	155	11.10	0.999	1-1000
acenaphtylene	152	153	150	12.07	0.997	1-1000
acenaphtene	154	153	155	12.48	0.998	1-1000
fluorene	166	167	164	13.80	0.998	1-1000
D10-phenanthrene	188	189	184	16.72	0.999	1-1000
phenanthrene	178	179	176	16.80	0.998	1-1000
anthracene	178	179	176	16.98	0.995	1-1000
fuoranthene	202	203	200	21.40	0.995	1-1000
pyrene	202	203	200	22.34	0.994	1-1000
terphenyl (ISTD)	230	228	215	23.23	1.000	200
retene	219	234	205	23.71	0.990	1-1000
benzo(b)fluorene	216	215	217	24.13	0.988	1-1000
benzo-naphtho-thiophene	234	235	232	26.59	0.991	1-1000
benzo(ghi)fluoranthene	226	227	224	26.78	0.991	1-1000
cyclopenta(cd)pyrene	226	227	224	27.65	0.976	1-1000
benzo(a)anthracene	228	229	226	27.69	0.985	1-1000
triphenylene	228	229	226	27.79	0.995	1-1000
chrysene	228	229	226	27.84	0.993	1-1000
benzo(b)fluoranthene	252	253	250	32.35	0.987	1-1000
benzo(j)fluoranthene	252	253	250	32.39	0.984	1-1000
benzo(k)fluoranthene	252	253	250	32.46	0.985	1-1000
benzo(e)pyrene	252	253	250	33.44	0.986	1-1000
benzo(a)pyrene	252	253	250	33.64	0.976	1-1000
D12-perylene	264	265	260	33.87	0.980	1-1000
perylene	252	253	250	33.97	0.978	1-1000
indeno(123cd)pyrene	276	277	274	38.19	0.977	1-1000
dibenzo(ah)anthracene	278	279	276	38.29	0.986	1-1000
dibenzo(ac)anthracene	278	279	276	38.30	0.976	1-1000
benzo(ghi)perylene	276	277	274	39.40	0.983	1-1000
anthanthrene	276	277	274	40.04	0.958	1-1000
coronene	300	301	298	48.67	0.982	1-1000

Table S2. Nitro-PAHs information (MRM, RT, linearity, calibration range)

Analyte	Quant MRM	Qual MRM	RT	linearity (R^2)	calibration range
	m/z	m/z	min		ng/mL
13C PCB 95 (ISTD)	337.9 > 267.9	339.9 > 267.9	11.11	1.00	10
1-nitronaphthalene	174 > 127.01	173 > 145	6.02	1.00	0.1-100
2-nitronaphthalene	173 > 127	174 > 127.01	6.41	1.00	0.1-100
3-nitroacenaphthene	200 > 153	199 > 169	9.90	1.00	0.1-100
5-nitroacenaphthene	199 > 169	200 > 153	10.42	1.00	0.1-100
2-nitrofluorene	211 > 164	212 > 195	11.81	1.00	0.1-100
9-nitroanthracene	223 > 193	223 > 178	12.14	1.00	0.1-100
9-nitrophenanthrene	223 > 167	223 > 178	13.23	1.00	0.1-100
3-nitrophenanthrene	223 > 176	223 > 193	14.01	1.00	0.1-100
2-nitrofluoranthene	247 > 201	248 > 202	18.10	0.99	0.1-100
3-nitrofluoranthene	247 > 217	247 > 189	18.12	0.99	0.1-100
1-nitropyrene	247 > 217	247 > 201	18.92	0.99	0.1-100
2-nitropyrene	247 > 201	248 > 202	19.26	0.99	0.1-100
7-nitrobenzoaanthracene	273 > 215	274 > 257	21.58	1.00	0.1-100
6-nitrochrysene	273 > 215	274 > 226	22.81	0.98	0.1-100
1,3-dinitropyrene	292 > 188	292 > 176	23.87	0.88	0.1-100
1,6-dinitropyrene	292 > 176	292 > 232	24.71	1.00	0.1-100
1,8-dinitropyrene	292 > 176	292 > 232	25.14	0.89	0.1-100
3-nitrobenzanthrone	245.1 > 217.2	246.1 > 218.2	25.14	0.93	0.1-100
6-nitrobenzoapyrene	297 > 239	297 > 224	26.94	0.99	0.1-100
1-nitronaphthalene-D7	181 > 133	181 > 134	6.00	0.99	10
2-nitrofluorene-D9	220 > 174	219 > 172	11.73	1.00	10
9-nitroanthracene-D9	233 > 215	232 > 214	12.08	0.99	10
3-nitrofluoranthene-D9	257 > 210	257 > 208	18.06	0.99	10
1-nitropyrene-D9	257 > 239	257 > 209	18.85	0.99	10
6-nitrochrysene-D11	285 > 235	285 > 238	22.71	1.00	10
6-nitrobenzoapyrene-D11	309 > 262	309 > 260	26.86	1.00	10

Table S3. Oxy-PAHs information (MRM, RT, linearity, calibration range)

Analyte	Quant MRM	Qual MRM	RT	linearity (R^2)	calibration range
	m/z	m/z	min		ng/mL
13C PCB 95 (ISTD)	337.9 > 267.9	339.9 > 267.9	11.11	1.00	10
1,4-naphthoquinone	158 > 102	159 > 103	4.75	0.98	0.1-100
naphthalene-1-aldehyde	156 > 128	157 > 128	5.33	1.00	0.1-100
dibenzofuran	168 > 139	169 > 140	5.40	0.99	0.1-100
9-fluorenone	180 > 152	181 > 153	7.30	1.00	0.1-100
6H-benzo[c]chromen-6-one	196 > 139	197 > 140	9.81	1.00	0.1-100
9,10-anthraquinone	208 > 152	209 > 153	10.03	0.97	0.1-100
9,10-phenanthroquinone	180 > 152	209 > 153	11.25	0.99	0.1-100
benzo-a-fluoren-11-one	230 > 202	231 > 203	15.48	0.99	0.1-100
benzo-b-fluoren-11-one	230 > 202	231 > 203	16.48	0.99	0.1-100
benzanthrone	230 > 202	231 > 203	17.62	1.00	0.1-100
benz(a)anthracene-7,12-dione	258 > 202	259 > 203	18.82	1.00	0.1-100
5,12-naphthacenequinone	258 > 202	259 > 203	20.30	1.00	0.1-100
6H-benzo[c,d]pyren-6-one	254 > 226	255 > 227	22.58	1.00	0.1-100
dibenzofuran-d8	176 > 146	177 > 147	5.38	0.99	10
9-fluorenone-d8	188 > 160	189 > 161	7.25	1.00	10
9,10-anthraquinone-d8	217 > 159	218 > 160	9.95	0.99	10

Table S4. Method performance parameters (% recovery average, % RSD)

Analyte	% recovery avg	% RSD
1,4-naphthoquinone	50	4
naphthalene-1-aldehyde	61	8
9-fluorenone	193	5
9,10-anthraquinone	93	2
1,4-anthraquinone	78	4
9,10-phenanthroquinone	71	4
benzo-b-fluoren-11-one	102	5
benzanthrone	100	2
benz(a)anthracene-7,12-dione	98	8
5,12-naphthacenequinone	93	10
2-nitronaphthalene	81	10
2-nitrofluorene	99	9
9-nitroanthracene	59	14
3-nitrophenanthrene	98	9
2-nitrofluoranthene	77	12
3-nitrofluoranthene	99	11
1-nitropyrene	96	11
1,3-dinitropyrene	68	6
1,6-dinitropyrene	67	7
1,8-dinitropyrene	65	9
naphthalne	55	14
biphenyl	73	11
acenaphthylene	66	12
acenaphthene	73	12
fluorene	93	11
phenanthrene	97	14
anthracene	94	10
fluroanthene	103	5
pyrene	103	4
retene	109	2
benzo(b)fluorene	112	3
benzo-naphtho-thiophene	101	3
benzo(ghi)fluoranthene	99	3
cyclopenta(cd)pyrene	78	4
benza(a)anthracene	98	3
triphenylene	97	3
chrysene	98	3
benzo(b)fluoranthene	94	5
benzo(j)fluoranthene	104	3
benzo(k)fluoranthene	99	3
benzo(e)pyrene	97	3
benzo(a)pyrene	98	2
perylene	94	3
indeno(123cd)pyrene	85	3
dibenzo(ah)anthracene	84	3
dibenzo(ac)anthracene	83	3
benzo(ghi)perylene	91	3
anthanthrene	88	13
coronene	99	5

Table S5. List of used chemicals and their purity

Chemical	Purity	Company
2-hydroxynaphthalene (13C6)	(13C6, 99%)	Cambridge Isotope Laboratories, Inc., Tewksbury, MA, U.S.A.
2-hydroxyfluorene (random-13C6)	(13C6, 99%)	Cambridge Isotope Laboratories, Inc., Tewksbury, MA, U.S.A.
3-hydroxyfluorene (13C6)	(13C6, 98%)	Cambridge Isotope Laboratories, Inc., Tewksbury, MA, U.S.A.
1-hydroxyphenanthrene (13C6)	(13C6, 99%)	Cambridge Isotope Laboratories, Inc., Tewksbury, MA, U.S.A.
2-hydroxyphenanthrene (13C6)	(13C6, 99%)	Cambridge Isotope Laboratories, Inc., Tewksbury, MA, U.S.A.
3-hydroxyphenanthrene (13C6)	(13C6, 98%)	Cambridge Isotope Laboratories, Inc., Tewksbury, MA, U.S.A.
4-hydroxyphenanthrene (13C6)	(13C6, 99%)	Cambridge Isotope Laboratories, Inc., Tewksbury, MA, U.S.A.
1-hydroxypyrene (13C6)	(13C6, 99%)	Cambridge Isotope Laboratories, Inc., Tewksbury, MA, U.S.A.
3-hydroxybenzo(a)pyrene (13C6)	(13C6, 98%)	Toronto Research Chemicals, Inc., North York, ON, Canada
1-hydroxynaphthalene	99%	Neochema GmbH., Bodenheim, Germany
2-hydroxynaphthalene	99%	Neochema GmbH., Bodenheim, Germany
2-hydroxyfluorene	98%	Neochema GmbH., Bodenheim, Germany
3-hydroxyfluorene	99%	Neochema GmbH., Bodenheim, Germany
1-hydroxyphenanthrene	98%	Neochema GmbH., Bodenheim, Germany
2-hydroxyphenanthrene	98%	Neochema GmbH., Bodenheim, Germany
3-hydroxyphenanthrene	99%	Neochema GmbH., Bodenheim, Germany
4-hydroxyphenanthrene	99%	Neochema GmbH., Bodenheim, Germany
1-hydroxypyrene	99%	Neochema GmbH., Bodenheim, Germany
3-hydroxybenzo(a)pyrene	95%	Sigma-Aldrich, Prague, Czech Republic
β-glucuronidase from *Escherichia coli*	Type IX-A, 2MU	Merck KG&A, Darmstadt, Germany
sodium acetate	p.a.	Sigma-Aldrich, Prague, Czech Republic
ammonium fluoride	p.a.	Sigma-Aldrich, Prague, Czech Republic
acetic acid	p.a.	Sigma-Aldrich, Prague, Czech Republic
methanol	LC/MS grade	Biosolve, Dieuze, France
formic acid	98%-100%	Merck KG&A, Darmstadt, Germany
dimethylsulfoxide	p.a.	Sigma-Aldrich, Prague, Czech Republic

Table S6. PAHs, oxy-PAHs, nitro-PAHs LOD and LOQ values in BAL samples

Analyte	LOD ng/mL	LOQ ng/mL
naphthalene	2.485	7.455
acenaphthylene	0.060	0.182
acenaphthene	0.035	0.105
fluorene	0.342	1.026
phenanthrene	1.078	3.233
anthracene	0.051	0.151
fluoranthene	1.096	3.287
pyrene	1.000	3.000
benzo(a)anthracene	0.041	0.125
chrysene	0.017	0.050
benzo(b)fluoranthene	0.085	0.255
benzo(k)fluoranthene	0.092	0.278
benzo(a)pyrene	0.109	0.328
indeno(123cd)pyrene	0.063	0.189
dibenzo(ah)anthracene	0.076	0.228
benzo(ghi)perylene	0.072	0.216
biphenyl	0.370	1.110
retene	0.151	0.453
benzo(b)fluorene	0.033	0.101
benzo-naphto-thiophene	0.026	0.080
benzo(ghi)fluoranthene	0.016	0.048
cyclopenta(cd)pyrene	0.056	0.169
triphenylene	0.016	0.048
benzo(j)fluoranthene	0.082	0.248
benzo(e)pyrene	0.082	0.246
perylene	0.100	0.300
dibenzo(ac)anthracene	0.076	0.228
anthanthrene	0.109	0.328
coronene	0.098	0.296
1-nitronaphthalene	0.005	0.015

Analyte	LOD ng/mL	LOQ ng/mL
2-nitronaphthalene	0.031	0.094
3-nitroacenaphthene	0.080	0.241
5-nitroacenaphthene	0.011	0.035
2-nitrofluorene	0.011	0.033
9-nitroanthracene	0.037	0.112
9-nitrophenanthrene	0.001	0.004
3-nitrophenanthrene	0.008	0.024
2-nitrofluoranthene	0.014	0.042
3-nitrofluoranthene	0.003	0.011
1-nitropyrene	0.013	0.041
2-nitropyrene	0.002	0.008
7-nitrobenzoaanthracene	0.023	0.071
6-nitrochrysene	0.002	0.008
1.3-dinitropyrene	0.001	0.003
1.6-dinitropyrene	0.009	0.029
1.8-dinitropyrene	0.002	0.006
3-nitrobenzanthrone	0.634	1.904
6-nitrobenzoapyrene	0.004	0.012
1.4-naphthoquinone	0.861	2.585
naphthalene-1-aldehyde	0.121	0.365
dibenzofuran	0.314	0.944
9-fluorenone	0.842	2.527
6h-benzo[c]chromen-6-one	0.580	1.741
9.10-anthraquinone	1.062	3.187
benzo-a-fluoren-11-one	0.038	0.113
benzo-b-fluoren-11-one	0.031	0.094
benzanthrone	0.046	0.138
benz(a)anthracene-7.12-dione	0.015	0.045
5.12-naphthacenequinone	0.005	0.016
6H-benzo[c.d]pyren-6-one	0.024	0.073

Table S7. OH-PAHs LOD and LOQ values in urine samples

Analyte	LOD ng/mL	LOQ ng/mL
1-hydroxynaphthalene	0.005	0.015
2-hydroxynaphthalene	0.006	0.019
2-hydroxyfluorene	0.006	0.018
3-hydroxyfluorene	0.006	0.018
2/3-hydroxyphenanthrene	0.006	0.018
9- hydroxyphenanthrene	0.070	0.020
1-hydroxyphenanthrene	0.006	0.018
4-hydroxyphenanthrene	0.006	0.018
1-hydroxypyrene	0.006	0.018
3-hydroxybenzo(a)pyrene	0.006	0.200
