# Supplementary material for: Polycyclic aromatic hydrocarbons and their metabolites in bronchoalveolar lavage and urine samples from patients with inhalation injury throughout their hospitalization: A prospective pilot study
Source: PLoS One. 2024 Aug 1;19(8):e0308163. doi: 10.1371/journal.pone.0308163 (PMC11293749; doi:10.1371/journal.pone.0308163)
Supplement: Attachment — Submitted filename: PONE-D-24-10533R1_Response to Reviewers_final.docx [file pone.0308163.s005.docx]

July 16^th^, 2024

PONE-D-24-10533R1

**Response to Reviewers (Editor)**

**Editor´s Comment 1:** After my personal, careful evaluation of your revised version, and of the response provided to the reviewers, I think that your manuscript would be suitable for publication provided that the following minor change is made:
- remove the reference 19 inserted at the request of one of the reviewers, which is very marginally related to the manuscript and does not introduce any substantial improvement to the manuscript.

**Journal Requirement 1:** Please review your reference list to ensure that it is complete and correct. If you have cited papers that have been retracted, please include the rationale for doing so in the manuscript text, or remove these references and replace them with relevant current references. Any changes to the reference list should be mentioned in the rebuttal letter that accompanies your revised manuscript. If you need to cite a retracted article, indicate the article’s retracted status in the References list and also include a citation and full reference for the retraction notice.

**Answer 1:** We removed the reference required by reviewer from the manuscript. We really appreciate your insight and suggestion. All references and quality of figures were checked.

Rabieimesbah A, Sobhanardakani S, Cheraghi M, Lorestani B. Concentrations, Source Identification and Potential Ecological and Human Health Risks Assessment of Polycyclic Aromatic Hydrocarbons (PAHs) in Agricultural Soils of Hamedan County, West of Iran. *Soil Sediment Contam Int J*. 2024;33: 482–506.

**Journal Requirements 2:** While revising your submission, please upload your figure files to the Preflight Analysis and Conversion Engine (PACE) digital diagnostic tool, <https://pacev2.apexcovantage.com/>. PACE helps ensure that figures meet PLOS requirements. To use PACE, you must first register as a user. Registration is free. Then, login and navigate to the UPLOAD tab, where you will find detailed instructions on how to use the tool. If you encounter any issues or have any questions when using PACE, please email PLOS at [figures@plos.org](mailto:figures@plos.org). Please note that Supporting Information files do not need this step.

**Answer 2:** Thank you for this recommendation. All figures from the main text have been uploaded to PACE tool and, thus, they should be meeting PLOS requirements.
